# Adult age-dependent differences in resting-state connectivity within and between visual-attention and sensorimotor networks

**DOI:** 10.3389/fnagi.2013.00067

**Published:** 2013-10-29

**Authors:** Christian Roski, Svenja Caspers, Robert Langner, Angela R. Laird, Peter T. Fox, Karl Zilles, Katrin Amunts, Simon B. Eickhoff

**Affiliations:** ^1^Research Center Jülich, Institute of Neuroscience and Medicine (INM-1, INM-2)Jülich, Germany; ^2^Institute of Clinical Neuroscience and Medical Psychology, Heinrich-Heine-UniversityDüsseldorf, Germany; ^3^Research Imaging Institute, University of Texas Health Science CenterSan Antonio, TX, USA; ^4^Department of Physics, Florida International UniversityMiami, FL, USA; ^5^Department of Psychiatry, Psychotherapy, and Psychosomatics, RWTH Aachen UniversityAachen, Germany; ^6^JARA-BRAIN, Jülich-Aachen Research AllianceJülich/Aachen, Germany; ^7^C. and O. Vogt Institute for Brain Research, Heinrich-Heine-University DüsseldorfDüsseldorf, Germany

**Keywords:** aging, fMRI, resting state, functional connectivity, MACM, functional systems

## Abstract

Healthy aging is accompanied by structural and functional changes in the brain, among which a loss of neural specificity (i.e., dedifferentiation) is one of the most consistent findings. Little is known, however, about changes in interregional integration underlying a dedifferentiation across *different* functional systems. In a large sample (*n* = 399) of healthy adults aged from 18 to 85 years, we analyzed age-dependent differences in resting-state (RS) (task-independent) functional connectivity (FC) of a set of brain regions derived from a previous fMRI study. In that study, these regions had shown an age-related loss of activation specificity in visual-attention (superior parietal area 7A and dorsal premotor cortex) or sensorimotor (area OP4 of the parietal operculum) tasks. In addition to these dedifferentiated regions, the FC analysis of the present study included “task-general” regions associated with both attention and sensorimotor systems (rostral supplementary motor area and bilateral anterior insula) as defined via meta-analytical co-activation mapping. Within this network, we observed both selective increases and decreases in RS-FC with age. In line with regional activation changes reported previously, we found diminished anti-correlated FC for inter-system connections (i.e., between sensorimotor-related and visual attention-related regions). Our analysis also revealed reduced FC between system-specific and task-general regions, which might reflect age-related deficits in top-down control possibly leading to dedifferentiation of task-specific brain activity. Together, our results underpin the notion that RS-FC changes concur with regional activity changes in the healthy aging brain, presumably contributing jointly to age-related behavioral changes.

## Introduction

Effective information processing depends on the integrity of communication between the different nodes of functional brain systems. Through normal aging, substantial changes within and between brain networks occur (for review see Eyler et al., 2011; Grady, 2012). For example, it has been shown, that older adults exhibit lower connectivity within task-relevant networks and greater connectivity outside the task-relevant networks (Daselaar et al., 2006; Dennis et al., 2008; St Jacques et al., 2009). Moreover, changes of regional brain activity were repeatedly observed in older adults (cf. Eyler et al., 2011; Grady, 2012). These neural changes are in line with behavioral studies reporting increased associations between sensory, sensorimotor, and cognitive functions in elderly participants (Lindenberger and Baltes, 1994; Baltes and Lindenberger, 1997; Schaefer et al., 2006).

In a previous functional magnetic resonance imaging (fMRI) study, we investigated functional activation patterns in the sensorimotor and the visual attention systems in a large sample of healthy subjects between 20 and 70 years of age (Roski et al., 2013). When testing for age-related effects across both functional domains (visual attention and sensorimotor control), we found reduced activation in several task-relevant regions as well as increased activation in regions that were less activated in younger adults. This effect holds substantial similarity to the repeatedly observed age-related process of dedifferentiation, i.e., a loss of neural specificity within distinct functional systems (for review see Reuter-Lorenz and Park, 2010). Little is known, however, about the aberrations in neural networks, i.e., inter-regional integration, underlying a dedifferentiation across different functional systems as observed in that previous fMRI study. This raises the question to which extent the interaction within and between functional systems becomes less differentiated as a consequence of healthy aging. In this context it should be noted that age-related changes in the interplay between visual attention processes and the sensorimotor system might provide additional insights into the physiological changes across multiple brain systems with healthy aging. In addition to providing a complementary, network-based perspective on age-related dedifferentiation across systems, improved knowledge of age-related changes in functional integration may also provide an important background for the assessment of network pathology caused by neurodegenerative disorders. For example, it is assumed that the cognitive deficits in Alzheimer's disease may be attributed to the disease's severe effects on functional networks marked by a profound disruption of functional connectivity (FC) within and across brain networks (for review see Delbeuck et al., 2003). In this context, however, it remains an open question whether similar age-related FC changes in connectivity between/within the sensorimotor and visual-attention systems may also be found in healthy aging, as suggested by the previously observed, dedifferentiated, recruitment pattern.

These considerations promoted the current analysis of age-related changes in functional integration between regions of the visual-attention and sensorimotor systems that were previously shown to feature reduced functional specificity with increasing age. Our aim was thus to address the changes in network interactions underlying the observed dedifferentiation across functional domains. This goal was pursued by investigating age-related alteration of task-independent, i.e., resting-state (RS), FC between regions of the visual-attention or sensorimotor systems (functional seeds) that show a dedifferentiated recruitment pattern with increasing age. In this context it should be noted that temporally correlated brain activity in spatially distinct regions (i.e., FC) may not only arise from direct interaction (Eickhoff and Grefkes, 2011). Rather, FC, particularly between different functional systems, may also be mediated by task-general regions interacting with regions from either system. The inclusion of these regions (in addition to the functionally defined seed regions) in the FC analysis should hence offer insights into the mechanisms underlying the change of network interactions between the brain systems in the elderly.

In the present study, we investigated changes of RS-FC between functional seeds that showed an age-related dedifferentiated recruitment pattern and task-general regions that are functionally related to them. The functional seeds were defined based on results from a previous fMRI study (Roski et al., 2013), whereas the task-general brain regions were identified via meta-analytic connectivity modeling (MACM; Eickhoff et al., 2010; Robinson et al., 2010). Once these task-general regions were defined, a two-step RS-FC network analysis was performed. In the first step, the task-independent connectivity pattern of all regions was analyzed in a large adult sample (*n* = 399). In the second step, age-related changes in task-independent FC for this set of seed regions were analyzed in the same sample. This approach of examining the RS-FC including task-general regions in a large sample of healthy subjects should provide insights into age-related changes in the functional coupling between brain regions involved in sensorimotor and visual attention processing.

## Materials and methods

The initial seed regions for the current connectivity analysis were provided by regions that showed a less differentiated neural activity pattern across the sensorimotor and the visual attention systems with age, i.e., an age-by-task interaction (Roski et al., 2013). As mentioned above, additional task-general regions (i.e., regions that consistently interact with each of these seed regions) were defined using MACM. The ensuing set of brain regions (seed regions from the fMRI study and task-general regions from the MACM analysis) were analyzed in a two-step RS-FC network analysis to unveil (i) the task-independent inter-regional FC within the combined set of brain regions, and (ii) to analyze age-related changes of FC within this network. All specified analyses are described in detail in the following sections.

### Definition of seed regions

#### Seed regions based on fMRI

**Figure 1:**
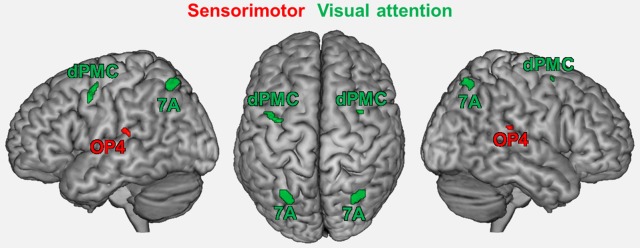
**Regions showing an age by task interaction as reported in a previous study (Roski et al., 2013)**. These regions represent the fMRI-based seed regions for the present study. Green color denotes regions associated with visual attention; red color denotes regions associated with sensorimotor processing. 7A, superior parietal lobule area 7A (cytoarchitectonically defined; Scheperjans et al., 2008a,b); dPMC, dorsal premotor cortex; OP4, area OP4 of the parietal operculum (cytoarchitectonically defined; Eickhoff et al., 2006a,b).

Seed regions were derived from a previous fMRI study on age-related changes in neural correlates of sensorimotor and visual attention processing (Roski et al., 2013). For the present study, only regions that showed an age-by-task interaction were included (Figure 1): bilateral area OP4 of the parietal operculum (Eickhoff et al., 2006a,b) showed a decrease in activation during a motor task (finger tapping) but an increase in activation during a visual attention task (target letter counting) in elderly participants. In contrast, bilateral superior parietal area 7A (Scheperjans et al., 2008a,b) and the rostral part of the dorsal premotor cortex (dPMC; cf. Brown et al., 2004; Ford et al., 2005; Amiez et al., 2006) showed the opposite pattern: an age-related decrease in activation during the visual attention task and an increase in activation during the motor task.

#### Task-general regions

Besides these functionally defined seeds, we also included task-general regions in the current analysis. We considered those regions to be task-general since, across tasks, they showed a consistent functional relation to each of the regions derived from the fMRI study. To identify these regions, we first mapped the task-dependent co-activation pattern for each of the above-mentioned seed regions using MACM. Subsequently, a conjunction analysis across the resulting co-activation maps using a minimum statistic approach revealed those regions that were functionally related to all fMRI-based seeds.

Here we used the BrainMap database (Laird et al., 2009, 2011; www.brainmap.org) to assess the co-activation pattern of each seed (Eickhoff et al., 2010), considering all experiments which reported stereotaxic coordinates from normal mapping studies (no interventions and no group comparisons) in healthy subjects using either fMRI or positron emission tomography (PET). These inclusion criteria yielded (at the time of analysis) ~7200 functional neuroimaging experiments. For each individual seed region (left/right OP4, 7A, DPMC) we proceeded as follows: first, we identified the 100 experiments in BrainMap that reported activation closest to it (see Table 1). Then we tested for convergence across (all) foci reported in these experiments using the revised version (Eickhoff et al., 2009) of the activation likelihood estimation (ALE) approach. Using random-effects inference, the ALE maps reflecting the convergence of co-activations with each seed region, were thresholded at *p* < 0.05 (cluster-level family wise error corrected; cluster-forming threshold: *p* < 0.001 at voxel level) and converted to Z-scores for visualization (see supplementary material Figure S1). As experiments were selected by activation close to the seed, highest convergence will be observed in the seed region (cf. Table 1). Significant convergence in other brain regions in turn indicates consistent co-activation over experiments and, hence, FC with the seed (Jakobs et al., 2012).

**Table 1:** **Location of seed regions and maximum distance to relevant foci within BrainMap**.

**Seed region**	**Peak coordinate (MNI: x,y,z)**	**Maximum distance (mm)**
7A left	−29, −66, 57	6.9
7A right	32, −66, 56	7.1
OP4 left	−44, −17, 12	7.3
OP4 right	50, −14, 11	7.4
dPMC left	−41, 2, 50	5.7
dPMC right	35, 6, 60	7.8

**Figure 2:**
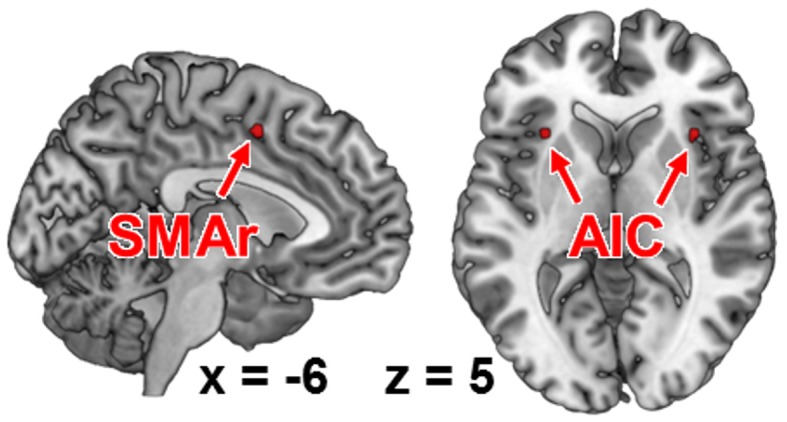
**Functional intersection of all co-activation maps of each fMRI-based seed region, as revealed by meta-analytic connectivity modeling**. These regions represent those brain areas that were functionally connected to all of our seed regions (i.e., task-general regions). AIC, anterior insular cortex; SMAr, rostroventral supplementary motor area.

To identify task-general regions for the subsequent RS-FC analysis, i.e., regions that were significantly co-activated with all seeds, we performed a conjunction analysis across the respective MACM results using the conservative minimum statistic (Nichols et al., 2005). This approach showed that the bilateral anterior insular cortex (AIC; MNI: −32, 20, 6; 36, 18, 4) was consistently co-activated with all seed regions (Figure 2). Also a region within the rostroventral supplementary motor cortex (SMAr; 2, 0, 56), rostrally bordering the pre-SMA and ventrally bordering the middle cingulate cortex (Palomero-Gallagher et al., 2008, 2009; Hoffstaedter et al., 2012), was consistently co-activated with each seed region. Both regions were hence defined to hold a task-general function across sensorimotor and visual attention processes.

#### Combined set of seed regions—fMRI-based seed regions and task-general regions

**Figure 3:**
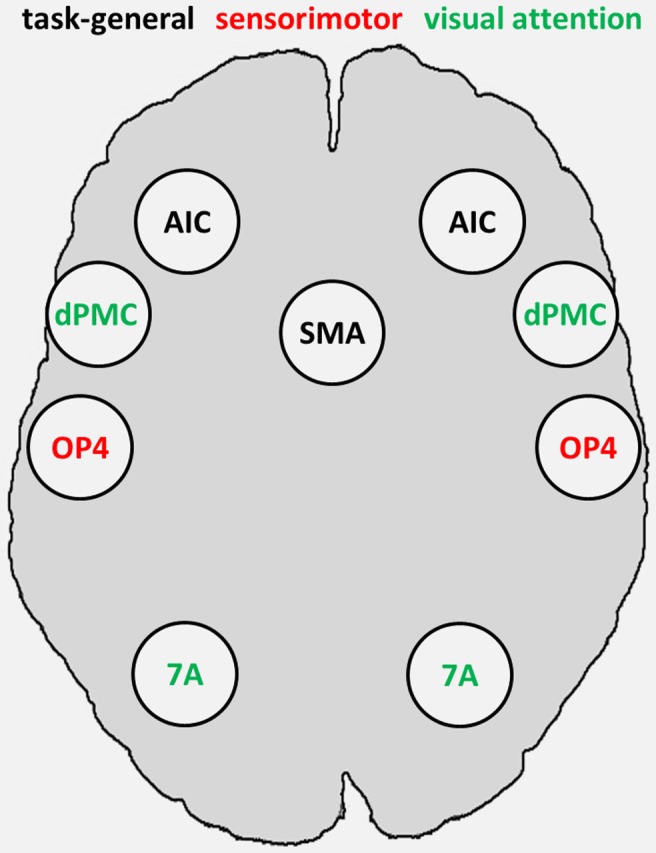
**Combined set of seed regions (schematic), comprising fMRI-based seed regions (visual attention and sensorimotor) derived from Roski et al. (2013) and task-general regions derived from meta-analytic connectivity modeling**. 7A, superior parietal lobule area 7A; AIC, anterior insular cortex; dPMC, dorsal premotor cortex; OP4, area OP4 of the parietal operculum; SMAr, rostroventral supplementary motor area.

The fMRI-based seed regions (visual attention and sensorimotor) and the MACM-derived task-general seed regions conjointly represented the combined set of our seed regions, comprising sensorimotor area OP4 and attention-related dPMC and area 7A, as fMRI-based seed regions, as well as SMAr and AIC as task-general regions (Figure 3).

### Resting-state analysis

An RS-FC network analysis was implemented to analyze the task-independent FC between these seed regions and its change with age for all possible connections between the combined set of seed regions (fMRI-based and task-general).

#### Sample

Inter-regional RS-FC for the combined set of seed regions was assessed by using RS fMRI data from 399 healthy volunteers, aged 18–85 years (*Mean* = 41.8, *SD* = 16.8, *Median* = 41, *IQR* = 29). All participants (46% female) were without any record of neurological or psychiatric disorders and gave their written informed consent to participate in the study. The data were contributed by four sites (see Table 2). Joint (re-)analysis of the data was approved by the local ethics committee of the Heinrich Heine University Düsseldorf.

**Table 2:** **Characteristics of the sample**.

**Source site**	***n***	**Mean age (range)**	**Sex: male (%)**	**Measurement parameters^a^**
RWTH University Hospital Aachen, Germany	47	36.5 (19–59)	46	3T/250/2.2/30/80°/3.1 × 3.1 × 3.1 mm^3^
	28	63.4 (55–72)	71	3T/270/2.2/30/90°/3.1 × 3.1 × 3.1 mm^3^
Research Center Jülich, Germany	51	28.3 (18–59	57	3T/250/2.2/30/90°/3.1 × 3.1 × 3.1 mm^3^
	100	45.1 (21–71)	48	3T/300/2.2/30/90°/3.1 × 3.1 × 3.1 mm^3^
ICBM, Montreal, Canada^b^	41	40.6 (19–78)	42	1.5T/256/2.0/50/90°/4.0 × 4.0 × 5.5 mm^3^
NKI, Rockland, NY, USA^b^	132	42.3 (18–85)	59	3T/260/2.5/30/80°/3.0 × 3.0 × 3.0 mm^3^

ICBM, International Consortium for Brain Mapping; NKI, Nathan S. Kline Institute.

a Measurement parameters: magnetic field strength of the scanner/number of acquired volumes/repetition time (in s)/echo time (in ms)/flip angle/voxel size.

b These data were selected from the datasets included in Biswal et al. (2010) and made publicly available via the 1000 Functional Connectomes Project.

#### Imaging and pre-processing

During scanning participants were instructed to let their mind wander but not to fall asleep which was confirmed by post-scan debriefing. For each subject the RS EPI images were acquired using blood-oxygen-level-dependent (BOLD) contrast (cf. Table 1). Image acquisition was preceded by dummy images allowing for magnetic field saturation which were discharged prior to further processing using SPM8 (www.fil.ion.ucl.ac.uk/spm). The EPI images were first corrected for head movement by affine registration using a two-pass procedure. For normalization the mean EPI images were segmented into gray matter, white matter and cerebrospinal fluid using the “unified segmentation” approach (Ashburner and Friston, 2005). The resulting parameters of a discrete cosine transform, which define the deformation field necessary to move subject data into MNI space, were then combined with the deformation field transforming between the latter and the MNI single-subject template. The ensuing deformation was subsequently applied to the individual EPI volumes which thereby were transformed into the MNI single-subject space and resampled at 1.5 mm isotropic voxel size. Finally, images were smoothed by a 5-mm FWHM Gaussian to meet requirements of the general linear model and compensate for residual anatomical variations.

#### Analysis

FC analyses may be influenced by several confounds such as physiological processes, e.g., fluctuations related to cardiac and respiratory cycles and in particular motion-related effects (Bandettini and Bullmore, 2008; Fox et al., 2009). In order to reduce spurious correlations, variance that could be explained by the following nuisance variables was removed from each voxel's time series (Reetz et al., 2012; Zu Eulenburg et al., 2012; Satterthwaite et al., 2013): (i) the six motion parameters derived from the image realignment, (ii) the first derivatives of the six motion parameters, (iii) mean gray matter, white matter and cerebrospinal-fluid signal per time point as obtained by averaging across voxels attributed to the respective tissue class in the SPM8 segmentation. All nuisance variables entered the model as first and second order terms. Following confound removal data was band pass filtered preserving frequencies between 0.01 and 0.08 Hz (Biswal et al., 1995; Greicius et al., 2003; Fox and Raichle, 2007). The time course of each seed region was then extracted for each subject as the first eigenvariate of all gray-matter voxels located within 5 mm of the peak coordinate. For each subject the time-series data of all seed regions was then cross-correlated to quantify the degree of FC between the seed regions. The ensuing pair-wise correlation coefficients were subsequently transformed into Fisher's Z scores. Statistically significant connectivity was assessed via one-sample *t*-tests (*p* < 0.05, corrected for multiple comparisons using the false discovery rate). Subsequently, the same Fisher-Z transformed correlation coefficients for each connection were rank-correlated with age to test for age-related changes in inter-regional coupling. The results of this correlation analysis were regarded significant if they passed a threshold of *p* < 0.05, corrected for multiple comparisons using the false discovery rate. Finally, we analyzed the connectivity in the 100 youngest and 100 oldest participants for all connections showing significant age-related changes, to corroborate the correlational findings. Moreover, this analysis permits a more detailed analysis of each age-related change of RS-FC to test, e.g., if an age-related FC increase represents a significant change from negative to positive FC or from negative to absent FC. The former pattern would imply an inversion of the RS communication (i.e., an increased functional interplay for relevant regions), while the latter would imply a loss of communication between brain regions.

## Results

### RS-FC within and between functional systems

**Figure 4:**
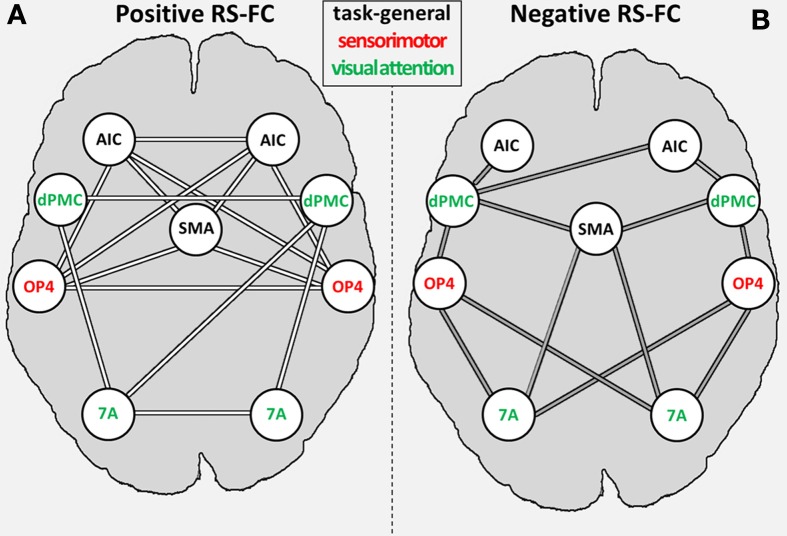
**General positive (A) and negative (B) inter-regional resting-state functional connectivity (RS-FC) for the combined set of seed regions**. Regions labeled in green are associated with the visual attention system; regions labeled in red are associated with the sensorimotor system [as indicated by the previous fMRI study (OP4, dPMC, and 7A; Roski et al., 2013)]; regions labeled in gray have no functional preference (i.e., task-general regions). 7A, superior parietal lobule area 7A; AIC, anterior insular cortex; dPMC, dorsal premotor cortex; OP4, area OP4 of the parietal operculum; SMAr, rostroventral supplementary motor cortex.

We found significant RS-FC for several connections within the combined set of seed regions. Significant positive coupling was found for: (i) SMAr with bilateral OP4 and bilateral AIC; (ii) left AIC with bilateral OP4; (iii) right AIC with bilateral OP4 and right area 7A; (iv) bilateral dPMC each with ipsilateral area 7A; (v) right DPMC with left area 7A; (vi) interhemispheric connections between bilateral regions AIC, dPMC, OP4, and area 7A (see Figure 4A). Noticeably, positive coupling was thus predominately found between regions with similar functional preferences (in the fMRI study), i.e., intra-domain connections (Figure 4A).

Significant negative coupling (i.e., anti-correlations) was found for the following connections: (i) SMAr with bilateral area 7A and right dPMC; (ii) bilateral OP4 to bilateral area 7A and dPMC; (iii) right AIC with bilateral dPMC and left AIC with left dPMC (see Figure 4B). Negative RS coupling was thus predominantly observable for inter-domain connections (Figure 4B), i.e., for regions with differing functional preferences in the original fMRI study.

### Age-related changes of RS-FC

**Figure 5:**
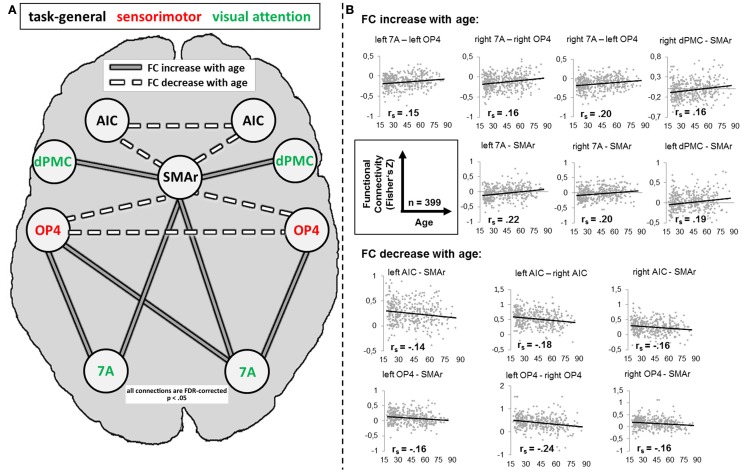
**(A)** Alteration of resting-state functional connectivity (FC) with age (schematic). White lines reflect significant positive resting-state FC between regions (independent of age); gray lines reflect significant negative resting-state FC between regions (independent of age). Dotted lines reflect a significant decrease [*p* < 0.05, corrected for multiple comparisons using the false discovery rate (FDR)] of FC with age; solid lines reflect a significant increase (*p* < 0.05, FDR-corrected) of FC with age. **(B)** Scatter plots for each age-affected interregional connection. Black line = linear regression line; *r_s_* = Spearman correlation coefficient: a negative value reflects a decrease of FC with age, whereas a positive value reflects an increase of FC with age. 7A, superior parietal lobule area 7A; AIC, anterior insular cortex; dPMC, dorsal premotor cortex; OP4, area OP4 of the parietal operculum; SMAr, rostroventral supplementary motor cortex.

For several connections, significant age effects on RS-FC were found (Figure 5). Specifically, RS-FC between SMAr and bilateral AIC and OP4, respectively, decreased with age. Also, the interhemispheric connectivity between left and right area OP4 and left and right AIC decreased with age. In contrast, an age-related increase of RS-FC was found for the bilateral connection of the DPMC and area 7A to the SMAr. Furthermore, area 7A bilaterally showed increased RS-FC to ipsilateral area OP4, whereas the right area 7A additionally showed an age-related increase of FC to the contralateral OP4. The supplementary extreme-group analysis in which we directly compared the 100 youngest and 100 oldest participants yielded significant differences for all connections that showed a significant association between RS-FC and age (see Table 3), corroborating the age-related correlational findings.

**Table 3:** **Age differences in intrinsic functional connectivity**.

**Connection**	**Mean *r*_young_**	**Mean *r*_old_**	**Correlation with age (*r*_s_)**
L 7A—L OP4	−0.164^***^	−0.087^***^	0.15
L 7A—SMAr	−0.111^***^	−0.004	0.22
R 7A—L OP4	−0.175^***^	−0.093^***^	0.20
R 7A—R OP4	−0.151^***^	−0.071^***^	0.16
R 7A—SMAr	−0.077^***^	0.019	0.20
L AIC—R AIC	0.515^***^	0.445^***^	−0.18
L dPMC—SMAr	−0.022	0.067^**^	0.19
R dPMC—SMAr	−0.041	0.042	0.16
L OP4—R OP4	0.472^***^	0.322^***^	−0.24
L OP4—SMAr	0.107^***^	0.025	−0.16
R OP4—SMAr	0.154^***^	0.078^***^	−0.17
SMAr—L AIC	0.289^***^	0.200^***^	−0.14
SMAr—R AIC	0.314^***^	0.212^***^	−0.16

Mean r_young_ and mean r_elderly_ denote group-averaged functional connectivity (FC) in the 100 youngest and 100 oldest participants, respectively. R, right; L, left; 7A, superior parietal area 7A; OP4, area OP4 of the parietal operculum; SMAr, rostroventral supplementary motor area; AIC, anterior insular cortex; dPMC, dorsal premotor cortex.

** significant at p < 0.001,

*** significant at p < 0.0001.

## Discussion

The present study investigated age-related differences in task-independent FC among a set of brain regions that was based on a previous fMRI study (Roski et al., 2013) to show a dedifferentiated recruitment pattern for visual-attention or sensorimotor demands (sensorimotor area OP4 and attention-related areas 7A and dPMC). This set was supplemented by task-general regions showing significant co-activation with all functional seeds across a broad range of tasks as identified by MACM analyses (SMAr and bilateral AIC). Together, these regions were used as seed regions for a network RS-FC analysis. This analysis demonstrated that regions belonging to the same functional domain show positive FC with each other, whereas regions belonging to different functional domains are anti-correlated with each other. The analysis of age-related changes in the RS-FC within the network revealed selective alterations of inter-regional connections, highlighting potential neural correlates of common age-related behavioral changes.

### Main effects of resting-state functional connectivity

The RS-FC analysis showed significant positive or negative correlations between RS activity of several seeds. When contrasting the RS-FC pattern with the task-dependent connectivity pattern (MACM), some connections showed corresponding FC, whereas other connections showed no FC congruency (for a detailed discussion see supplementary material). Positive RS-FC was predominantly observed between brain regions associated with similar functional systems (Figure 5A), whereas negatively correlated RS-FC was predominantly observed between regions belonging to different functional systems (see Figure 5B). In other words, RS activity in regions with similar functional characteristics appears to be positively correlated, whereas RS activity in regions with different functional properties is negatively correlated. In summary, the (original) functional distinction of the combined set of seed regions was well corroborated by its RS-FC pattern. Our results are thus in line with previous studies showing temporal correlations across functionally related areas, thereby forming RS networks that mirror task-related functional systems (De Luca et al., 2006; Smith et al., 2009; Biswal et al., 2010).

In turn, we found that negative FC was predominantly found between regions from different domains. These results underline an intrinsic, functionally driven organization of the human brain and are supported by studies showing that regions with apparently opposing functionality are negatively correlated in their RS-FC (Greicius et al., 2003; Roskies et al., 2013; Schlegel et al., 2013).

### Age-dependent differences in RS-FC

First of all, it is noteworthy that not all connections showed age-related changes, arguing against a general and unspecific age-related decline in the task-independent functional coupling between brain regions. Instead, it appears that changes across the lifespan occur selectively. Connections that showed age-related changes, however, mostly featured reductions of functional correlations, as both positive and negative RS-FC moved closer to zero with age. In other words, the task-independent functional correlation (i.e., negative or positive RS-FC) within and between the present networks appears to be diminished in older adults (see Figure 5B) making the network structure less distinct. According to the “disconnection” hypothesis proposed by O'sullivan et al. (2001), such functional “disruptions” in a network are associated with deteriorated white matter integrity and poorer cognitive performance across several functional domains. In line with this, a decreased FC *within* a distinct network, i.e., the default-mode network (Andrews-Hanna et al., 2007; Tomasi and Volkow, 2012), which is relevant for internally directed mental states including remembering, planning, and related cognitive functions (Greicius et al., 2003; Fransson, 2005; Buckner and Carroll, 2007) was demonstrated with age. In contrast to this, our findings indicate less RS communication *between* different task-related networks (i.e., visual-attention and sensorimotor networks). Nevertheless, we also found a few connections showing an increase in FC. The healthy aging brain is thus not only subject to functional decline but rather responds selectively to presumable structural or biochemical neuronal changes in the elderly. Again, this finding is particularly important in that it underlines that connectivity is not reduced *per se*, which may potentially be deemed to reflect systematic confounds. Rather, we found decreased (closer to zero), increased, or unchanged connections in our large sample.

#### Age-dependent differences in RS-FC within the sensorimotor system

The present results showed an age-related reduction of the task-independent interhemispheric connection of the left and right area OP4. This region is assumed to play a role in sensorimotor integration processes, such as incorporating sensory feedback into motor actions (Rizzolatti and Wolpert, 2005; Halsband and Lange, 2006) and tactile object recognition and manipulation (Inoue et al., 2002; Wasaka et al., 2005). As a consequence of the decreased interhemispheric FC for this region, a diminished communication within this “sensorimotor feedback” system may develop in advanced age. A study on age-related behavioral slowing in sensorimotor tasks, suggesting a dysregulation of sensorimotor processing (Yordanova et al., 2004), corroborates this assumption. In line with this, increased age is associated with slower performance in speeded motor tasks (Salthouse, 2000). Finally, even in simple tasks (i.e., auditory and visual choice reactions), age-related changes within the sensorimotor system appear to affect performance (Yordanova et al., 2004). Although area OP4 represents only a single node within the sensorimotor system, the altered task-independent coupling for this regions may indicate a neural correlate of the above-mentioned behavioral difficulties observed in older adults.

#### Age-dependent differences in RS-FC between visual-attention and sensorimotor-related regions

Age-related increases in RS-FC were observed for inter-system connections, i.e., between sensorimotor-related and visual attention–related regions (OP4—7A; see Figure 5). Importantly, RS-FC showed an anti-correlated pattern between those regions in the young subsample (Figure 4B). Thus, in young adults, task-independent neural activity in OP4 is accompanied by deactivation of area 7A and vice versa. This anti-correlation appears to be diminished in older adults, as RS-FC increased with age, approaching zero (see Figure 5). In other words, switching or mutual suppression (both of which would result in anti-correlation) between both sensorimotor and visual-attention network activity seems to be deteriorated in advanced age, potentially resulting in less distinct processing. This effect corroborates the fMRI study the functional seed regions were derived from (Roski et al., 2013). In that study we observed less differentiated task-dependent neural activity within areas 7A and OP4 in older adults, i.e., their activation patterns became less specific with age. Hence, the age-related changes of RS-FC observed here might reflect the previously demonstrated age-related alteration of a task-dependent activation pattern. Changes of intrinsic connectivity may hence be considered as a potential predictor for changes in task-dependent activation.

#### Age-dependent differences in RS-FC between task-specific regions and task-general seeds

Decreased RS-FC was observed between OP4 and the task-general SMAr, suggesting reduced communication between these regions with age. A similar effect was found for the attention-related area 7A. Here RS-FC was negative in the young subsample (see Table 3), the age-related FC increase (toward zero) reflects a loss of this anti-correlation, i.e., again a reduced communication. In other words, with age SMAr shows a less distinct connectivity with sensorimotor and visual attention related regions. In contrast, we found no significant age-related changes in RS-FC between the second task-general region (i.e., AIC in each hemisphere), and the task-specific regions.

The AIC is known to be a highly integrative region with relevance for the processing of somatosensory, cognitive and social-emotional information (Kurth et al., 2010). Likewise, the SMA is known to integrate neural information relevant for the internal generation of movements (Picard and Strick, 1996; Jenkins et al., 2000; Thickbroom et al., 2000; Crosson et al., 2001; Weeks et al., 2001; Cunnington et al., 2002). In line with this, we here identified the task-general regions SMAr and AIC by significant task-based FC to all of the fMRI-based seeds. Hence, both regions seem to subserve higher-order cognitive processes interacting with both sensorimotor and visual attention specific regions. In their influential study, Dosenbach et al. (2006) proposed a “core system for the implementation of task sets.” In that study, SMA and bilateral AIC were shown to be associated with the initiation and the maintenance of mental task sets. Notably, the present task-general regions closely correspond to this “core system” found by Dosenbach and colleagues. In another line of studies it was shown that regions in a very similar position as our task-general SMAr and AIC do not respond in a task-specific manner but rather to the degree of personal salience across tasks (e.g., Craig, 2002; Curtis and D'Esposito, 2003; Kerns et al., 2004). This “salience network” is thought to integrate highly processed sensory data with visceral, autonomic, and hedonic information (Damasio, 2000), so that the organism can decide what to do (or not to do) next. The reduced RS-FC with age between SMAr and the task-specific regions may hence imply difficulties for older adults to initiate, maintain, and switch activation in task-relevant functional systems. At the behavioral level, such difficulties were demonstrated for older adults during global task switching (Wasylyshyn et al., 2011) and dual-tasking (Verhaeghen et al., 2003; Just et al., 2008), that is, in situations where different mental task sets have to be constantly (re-)initiated or simultaneously maintained. The demonstrated age-related reduction in RS-FC between task-set control regions and task-specific regions (related to visual attention or sensorimotor processing) may reflect these difficulties at the neural level.

Moreover, our results also indicated a decreased functional coupling within the task-general network, i.e., between the SMAr, left and right AIC. As mentioned before, these regions represent basic nodes within a network assumed to be involved in task-switching and dual-tasking processes. The reduced intercommunication of these regions in older adults strengthens our above interpretation that intrinsic age-related changes possibly mediate task-switching and dual-tasking difficulties in older adults. In line with this, increased task-switching costs were interpreted in terms of an age-related impairment in the ability to internally differentiate among task sets (Keith et al., 2004).

In summary, we found reduced task-independent communication within the task-general regions as well as between the task-general region SMAr and the functional seed regions. The reduced communication within the task-general regions may reflect difficulties for older adults during task-switching and dual-tasking. Moreover, the reduced communication to the functional seed regions (sensorimotor and visual attention) may indicate more task specific impairments for older adults. In line with this, the interaction of the visual-attention and sensorimotor systems seems to be deteriorated in older adults (Szturm et al., 2013), possibly provoked by a deteriorated communication with the task-set system (task-general regions).

At the neural level, these intrinsic changes of intercommunication within and between the task-set system and functional seed regions might also explain the reduced distinctiveness of task-independent brain activity in the two functional systems (sensorimotor and visual attention), as found in our former study (Roski et al., 2013). In particular, the interplay of both systems may be controlled less precisely, potentially resulting in less distinct regional activations of task-specific brain regions.

Finally, an increased RS-FC with age was observed for the connection between the visual-attention-related dPMC and the task-general SMAr. In contrast to the decreased communication between the attention-related area 7A and task-general SMAr, this age-related increase reflects an enhancement of communication for this connection. In line with this, it was shown that older (vs. younger) adults often recruit more frontal regions to successfully perform a visual attention task (Ansado et al., 2012; Li et al., 2013). Our findings of decreased FC between 7A and SMAr and increased FC between dPMC and SMAr thus reflects that the communication between the more posterior area 7A and SMAr declines with age, whereas the communication between the more anterior dPMC and SMAr is enhanced. This effect may reflect an intrinsic posterior-to-anterior shift in aging (PASA; cf. Davis et al., 2008) for cognitive control within the visual attention system and, moreover, demonstrates that the PASA-effect, previously reported for regional brain activity, may also extend to RS-FC.

### Limitations and future directions

First, the current cross-sectional study offers the advantages of a large sample. However, some drawbacks have to be mentioned. Although a relation between age and changes in the interregional connection is clear, the causality between both is not positively determinable. In other words, there is no information on which variable caused the other. Moreover, we cannot completely exclude that additional variables, e.g., structural or neurochemical alterations, may influence the findings of the current study. In future research, the combination of structural MRI (e.g., diffusion tensor imaging), functional MRI, and neurochemical measurements, may extend the understanding of the causalities on FC changes in the aging brain. Second, since the analyzed functional networks reflect only parts of the visual-attention or sensorimotor system that showed an age-related loss of neural specificity, inferences from our results on age-related RS-FC changes between entire functional networks have to be regarded with caution. Nevertheless, our study indicates that healthy aging is associated with task-independent connectivity changes within and across task-specific brain network nodes. Furthermore, the current sample was derived by four different sites. Although all participants were screened for psychiatric and neurological disorders, the threshold for sub-clinical cognitive impairments may vary between them. Hence, the sample may contain some patients with sub-clinical symptoms. Finally, in the current sample we were unable to relate individual FC parameters to performance. Further studies should hence combine performance measurements with neuroimaging during task performance and task-free states in the same participants. This would permit investigating more direct relations of different age-related neural changes, their interdependencies, and their association with performance (see, e.g., Andrews-Hanna et al., 2007; Madden et al., 2010; Schulte et al., 2011).

### Conclusions

The present study corroborates the notion that RS activity reflects the functional organization of the human brain (De Luca et al., 2006; Biswal et al., 2010), as RS activity in regions with similar functional characteristics was positively correlated, whereas RS activity in regions with different functional properties was negatively correlated but also revealed anti-correlation between task-specific and (co-activated) task general regions. Second, age-related changes in network FC seem to be connection-specific, as not all RS connections were affected, and changes comprised both increases and decreases in RS-FC. Third, the majority of the observed age-related changes indicated a reduction of communication in the aging brain, as both correlations and anti-correlations were attenuated. Task-general regions, presumably relevant for the implementation and maintenance of task sets, showed reduced interregional RS-FC, in line with well-known difficulties of older adults in task-switching or dual-tasking. Furthermore, the communication between system-specific brain regions and the global task-set system seems to be intrinsically deteriorated, potentially leading to less differentiated regional brain activity during visual-attention and sensorimotor tasks, respectively (Roski et al., 2013). Finally, an age-related posterior-to-anterior shift was observed for the RS connectivity between areas of the visual attention system, in line with the PASA theory (Davis et al., 2008). In conclusion, our findings demonstrate that previously observed behavioral and functional brain activity changes concur with intrinsic FC changes in the healthy aging brain.

### Conflict of interest statement

The authors declare that the research was conducted in the absence of any commercial or financial relationships that could be construed as a potential conflict of interest.

## Supplementary material

Figure S1 **Visualization of the co-activation map for each task-specific seed region as revealed by meta-analytic connectivity modeling (MACM)**. L, left; R, right; DPMC, dorsal premotor cortex; 7A, superior parietal area 7A; OP4, area OP4 of the parietal operculum.
