# Supplementary figures and images for: Adult age-dependent differences in resting-state connectivity within and between visual-attention and sensorimotor networks
Source: Front Aging Neurosci. 2013 Oct 29;5:67. doi: 10.3389/fnagi.2013.00067 (PMC3810651; doi:10.3389/fnagi.2013.00067)

# Co-activation maps (MACM)

## dPMC

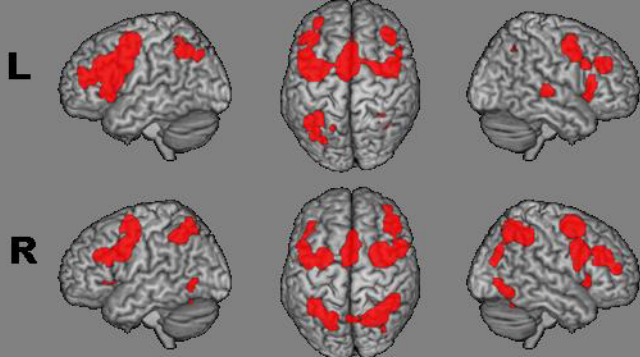

## 7A

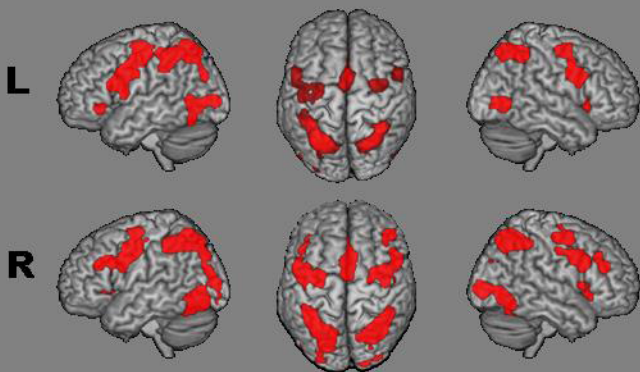

## OP4

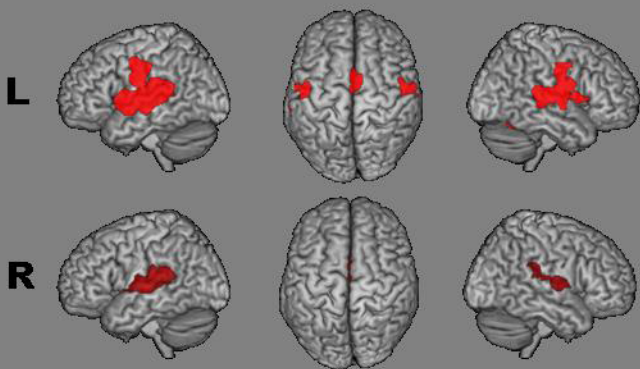

Supplement: Figure S1 — Visualization of the co-activation map for each task-specific seed region as revealed by meta-analytic connectivity modeling (MACM). L, left; R, right; DPMC, dorsal premotor cortex; 7A, superior parietal area 7A; OP4, area OP4 of the parietal operculum. [file Presentation1.PDF]
